# Dietary fiber, gut microbiota, and host immunity: the immunomodulatory roles of short-chain fatty acids

**DOI:** 10.3389/fnut.2026.1941474

**Published:** 2026-10-23

**Authors:** Xiaoli Hou, Yan Li, Weiwei Mu, Huiqin Wang, Shaoping Ji

**Affiliations:** Medical School of Health, Zhengzhou Health College, Zhengzhou, Henan, China

**Keywords:** gut microbiota, gut-organ axes, immune-regulation, inflammation, SCFAs

## Abstract

Short-chain fatty acids (SCFAs) are a class of key metabolites produced by the gut microbiota through the fermentation of indigestible components such as dietary fiber. They primarily include acetate, propionate, and butyrate. In recent years, a large body of research has revealed that SCFAs are not only an important energy source for colonic epithelial cells but also key signaling molecules linking the gut microbiota to the host immune system. SCFAs play a central role in immune regulation. This review summarizes the production and characteristics of SCFAs. It explores the multifaceted roles of SCFAs in regulating immune homeostasis and inflammatory responses. Through key mechanisms such as activation of G protein-coupled receptors (GPCRs), inhibition of histone deacetylases (HDACs), and metabolic reprogramming, SCFAs broadly regulate the differentiation, function, and inflammatory responses of immune cells, maintain intestinal barrier integrity, and induce an anti-inflammatory state. At the same time, the manuscript analyzes the molecular mechanisms by which SCFAs contribute to inflammatory bowel disease, metabolic disorders, tumor immunity, and neuro-immune diseases through multi-organ pathways such as the “gut-liver axis” and the “gut-brain axis”. In addition, this article summarizes treatment strategies targeting SCFAs, including dietary interventions, probiotics, prebiotics, and fecal microbiota transplantation. It also discusses the current challenges in this field and outlines future directions, with the aim of providing new insights for the prevention and treatment of immune-related diseases.

## 1 Introduction

In recent years, the interaction between the gut microbiota and the host immune system has become a hot topic of research in the biomedical field (1, 2). As a key metabolic link between the two, SCFAs have attracted sustained attention due to their extensive and profound immunomodulatory functions (3). SCFAs are primarily produced by the gut microbiota through the fermentation of dietary fiber (4–6). They are the key metabolic products of microbial fermentation and the most abundant microbial metabolites in the colonic lumen (7, 8). They primarily consist of acetate, propionate, and butyrate (7, 9). They serve not only as an important energy source for colonic epithelial cells, but are also widely recognized as key mediators in maintaining intestinal homeostasis and host health (10). Extensive evidence indicates that SCFAs play indispensable roles in regulating intestinal barrier function, maintaining immune homeostasis, and shaping innate and adaptive immune responses (3).

In the gut, SCFAs establish an anti-inflammatory, tolerant immune microenvironment through multiple mechanisms. They promote the differentiation of regulatory T cells (Tregs), enhance immunoglobulin A (IgA) secretion, and modulate the function of dendritic cells and macrophages (11, 12). They also maintain mucosal immune homeostasis by influencing inflammasome activation and lymphocyte proliferation (7, 13). From a molecular mechanism perspective, the immunomodulatory effects of SCFAs are achieved through three pathways. As ligands for GPCRs, they activate downstream signal transduction. They inhibit HDACs activity, thereby influencing the epigenetic modifications of immune-related genes. As energy substrates, they reshape the metabolic state of immune cells (4, 14).

Notably, the effects of SCFAs extend far beyond the gut (15). They can be absorbed into the bloodstream. Through the circulatory system, they affect distant tissues and organs, serving as key chemical mediators in communication along the “gut-organ axis” (16). In the liver, SCFAs regulate lipid metabolism and inflammatory responses through pathways such as PPAR-γ and AMPK (17). In the brain, they participate in modulating microglial function, neuro-inflammation, and blood-brain barrier integrity, serving as a key link in gut-brain axis communication (18, 19). Furthermore, SCFAs regulate immune homeostasis in the lungs, kidneys, oral cavity, and even visual organs (20). Consequently, SCFA dysregulation is closely associated with various diseases, including inflammatory bowel disease, metabolic syndrome, systemic autoimmune diseases, neurodegenerative disorders and tumors (21–23).

Given these critical functions, SCFAs and their signaling pathways have emerged as potential targets for intervening in various immune-related diseases. However, existing evidence also suggests that the role of SCFAs in various pathophysiological contexts is complex and context-dependent. In both vivo and vitro studies, SCFAs have demonstrated seemingly contradictory immunomodulatory effects: both anti-inflammatory and pro-inflammatory (24). What factors determine this functional switch? This paper analyzes how SCFA concentration, target cell types, the local microenvironment, and disease state collectively shape the immunomodulatory effects of SCFAs. It also explores gaps in the existing evidence and future research directions, thereby providing a theoretical basis for studying the mechanisms of relevant diseases and developing precision intervention strategies.

## 2 Production and characteristics of SCFAs

### 2.1 Sources of SCFAs

SCFAs are a class of organic fatty acids with fewer than atoms (25). They primarily include acetate, propionate, butyrate, and their branched-chain forms, such as isobutyrate and isovalerate (26). As core metabolites produced by gut microbiota fermentation of dietary fiber, SCFAs form pivotal nodes in the host-microbiota co-metabolism network. Their generation and characteristics directly determine the diversity and complexity of subsequent immune regulatory functions (27) (Table 1A).

**Table 1A:** Chemical and metabolic characteristics of SCFAs.

Feature	Acetate	Propionate	Butyrate
Structural formula	CH_3_COOH	CH_3_CH_2_COOH	CH_3_CH_2_CH_2_COOH
Molecular weight	Lowest among the three core SCFAs	Intermediate	Highest among the three core SCFAs
Water solubility	Highly water-soluble, efficiently absorbed in the intestine	Moderate water solubility	Relatively low water solubility
Blood stability	Diffusing readily into the bloodstream	Highly stable in the portal vein	——
Intestinal absorption	Efficiently absorbed	Minimal metabolic loss during intestinal absorption	——
Primary metabolic fate	Reaching systemic circulation; serving as key signaling molecule linking gut to distal adipose tissue	Regulating hepatic immune microenvironment and metabolic inflammation	Primary energy source for intestinal epithelial cells; prioritizing maintenance of intestinal barrier integrity
Major acid-producing bacterial communities	*Bifidobacterium, Lactobacillus, Prevotella, Bacteroides*	*Clostridium, Roseburia, Veillonella, Desulfovibrio, Bacteroides, Phascolarctobacterium, Dialister*	*Roseburia hominis, Faecalibacterium, Eubacterium, Clostridium, Ruminococcus, Bifidobacterium, Akkermansia,muciniphila, Bacteroides*
References	(28, 29)	(9, 30, 31)	(13)

There are two sources of SCFAs in the host. Direct dietary intake, with small amounts present in dairy products and vegetable oils, and indirect production through fermentation of undigested dietary fiber by gut microbiota (27). SCFAs from direct dietary sources are extremely limited in quantity, far insufficient to support their extensive roles in immune regulation (27). The primary source of SCFAs in the true sense is the anaerobic fermentation process carried out by colonic microbiota (8). This origin pattern holds significant physiological importance. It tightly couples SCFAs generation with the host's dietary structure and gut microbiota composition. This context-dependent nature serves as the logical starting point for understanding the functional variability of SCFAs. Differences in SCFAs production across individuals and pathological states likely stem from dysregulation within the upstream “diet-microbiota” axis.

### 2.2 Primary types and chemical properties of SCFAs

Although the SCFA family comprises numerous members, acetate, propionate, and butyrate are the three most abundant and extensively studied types (32). They are all short-chain saturated carboxylic acids. This simple structure endows them with unique biological properties. They can serve as energy substrates in cellular metabolism, act as signaling molecules interacting with specific receptors, and function as regulators of epigenetic modifications to influence gene expression (25). In current research, SCFAs are often considered a collective term for acetate, propionate, and butyrate. However, this simplification may obscure the functional specificity among different monomers. This issue requires further investigation in future studies.

### 2.3 Metabolism and absorption/transport of SCFAs

The metabolic pathways of SCFAs after generation form the basis for their biological functions. First, SCFAs are an important energy source for intestinal epithelial cells (IECs), with butyrate playing a particularly prominent role. Approximately 70% to 90% of butyrate is absorbed by colonic cells and serves as a preferred fuel source. It provides energy through β-oxidation to maintain the health and integrity of the intestinal epithelium (33). Second, SCFAs not locally utilized enter the bloodstream via specific transporters (such as mono-carboxylate transporter 1 and sodium-coupled mono-carboxylate transporter 1), enabling delivery to distant organs (16).

The metabolic fate of SCFAs is significantly dependent on carbon chain length. Butyrate is primarily utilized locally by colon cells. Propionate is mostly absorbed in the liver for gluconeogenesis. Acetate, on the other hand, enters the systemic circulation influence peripheral tissues (32). Understanding this metabolic diversion pattern is fundamental to deciphering SCFA signaling patterns within the “gut-organ axis”.

### 2.4 Diversity of SCFA functional profiles

Existing research indicates that SCFAs play multiple roles in host health and disease. First, as the primary energy source for colonic cells, SCFAs indirectly safeguard intestinal barrier integrity by sustaining epithelial metabolic activity (34). Second, SCFAs modulate the differentiation, activation, and effector functions of gut-associated immune cells (25) (Figure 1) (Table 1B). This regulation can be achieved either through direct action on immune cells or indirectly by influencing epithelial cell function. Third, SCFAs serve as precursors for carbohydrate and lipid synthesis and participate in the regulation of systemic energy homeostasis (35). Fourth, some studies suggest SCFAs can inhibit tumor cell proliferation and induce apoptosis, although their mechanisms of action and *in vivo* relevance require further elucidation (25).

**Figure 1:**
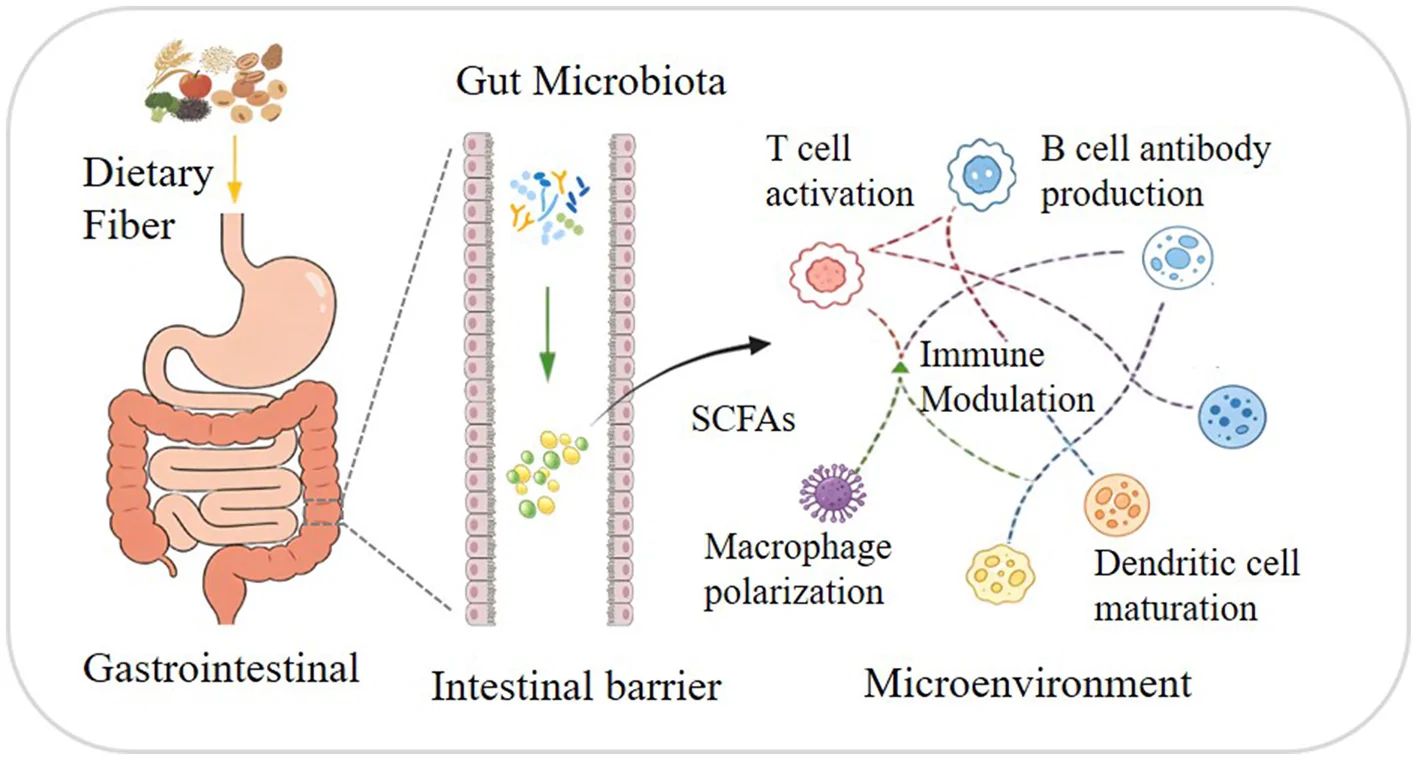
Regulatory effects of SCFAs on immune cells.

**Table 1B:** Functional specificity of SCFAs in immune regulation.

Immune cell type	Acetate	Propionate	Butyrate
Macrophages	—	Inhibiting M2 macrophage polarization; Reducing allergic airway inflammation	Inhibiting M2 polarization pathway; Enhancing antimicrobial effects via HIF-1α upregulation; suppresses IL-1β, IL-6, IL-10 production in myeloid cells
Dendritic cells (DCs)	Inhibiting IL-6 and TNF-α production in plasmacytoid DCs	InhibitingTh2 immune response initiation by DCs; Suppressing IL-6 and TNF-α production in myeloid and plasmacytoid DCs	Suppressing IL-6 and TNF-α production in myeloid and plasmacytoid DCs
T cells	Reducing TNF-α production in activated T cells	Suppressing IFN-γ, IL-17, IL-13, IL-10 production; Inhibiting CCR9/CCR10 expression; Promoting Treg differentiation	Promoting Treg and IL-10-producing Th1 cell differentiation; context-dependently regulates Th17 development; Suppressing IFN-γ, IL-17, IL-13, IL-10 production; Inhibiting CCR9/CCR10 expression; Enhancing CD8+ T cell IFN-γ production and memory formation
B cells	Modulating Ig production and class-switching	Suppressing Aicda and Prdm1 expression via HDAC inhibition; Inhibiting T-dependent and T-independent antibody responses	Suppressing Aicda and Prdm1 expression via HDAC inhibition; Inhibiting T-dependent and T-independent antibody responses; Suppressing autoantibody production in lupus models
Key functional signature	Higher concentrations in gut; major role in peripheral lipid synthesis and systemic immune regulation	Dual activity: GPCRs activation + weak HDACs inhibition; Regulating hepatic immune microenvironment	Strongest HDACs inhibitory activity among three core SCFAs; central role in epigenetic regulation, anti-inflammation, and immune homeostasis
Disease model evidence	Attenuating ZIKV-induced inflammatory responses via FFAR2/GPR43	Reducing allergic airway inflammation in asthma models	Alleviating asthma airway inflammation; attenuates cirrhotic portal hypertension via HDAC3 inhibition; Enhancing MSLN CAR-T cell antitumor activity; Alleviating muscle atrophy via autophagy inhibition and metabolic reprogramming
References	(31)	(9, 30)	(36–38)

As key metabolic products of gut microbiota, the production of SCFAs is dynamically regulated by the diet-microbiota axis. Their properties confer multifunctional roles ranging from energy substrates to signaling molecules. However, a deeper understanding of this field must go beyond a mere descriptive analysis of “multi-functionality”. Instead, it must focus on a detailed analysis of the functional specificity of different monomers, the dynamic regulatory mechanisms of SCFAs production and metabolism, and concentration-effect relationships.

## 3 Regulatory effects of SCFAs on immune cells and molecular mechanisms

SCFAs are key signaling molecules linking the gut microbiota to the host immune system (39, 40). There are three core and overlapping molecular pathways: activation of GPCRs (primarily GPR41/FFAR3, GPR43/FFAR2, and GPR109A) (41–43) (Figure 2), inhibition of HDACs (primarily Class I and Class II) (3) (Figure 3), and reprogramming cellular energy metabolism (44, 45).

**Figure 2:**
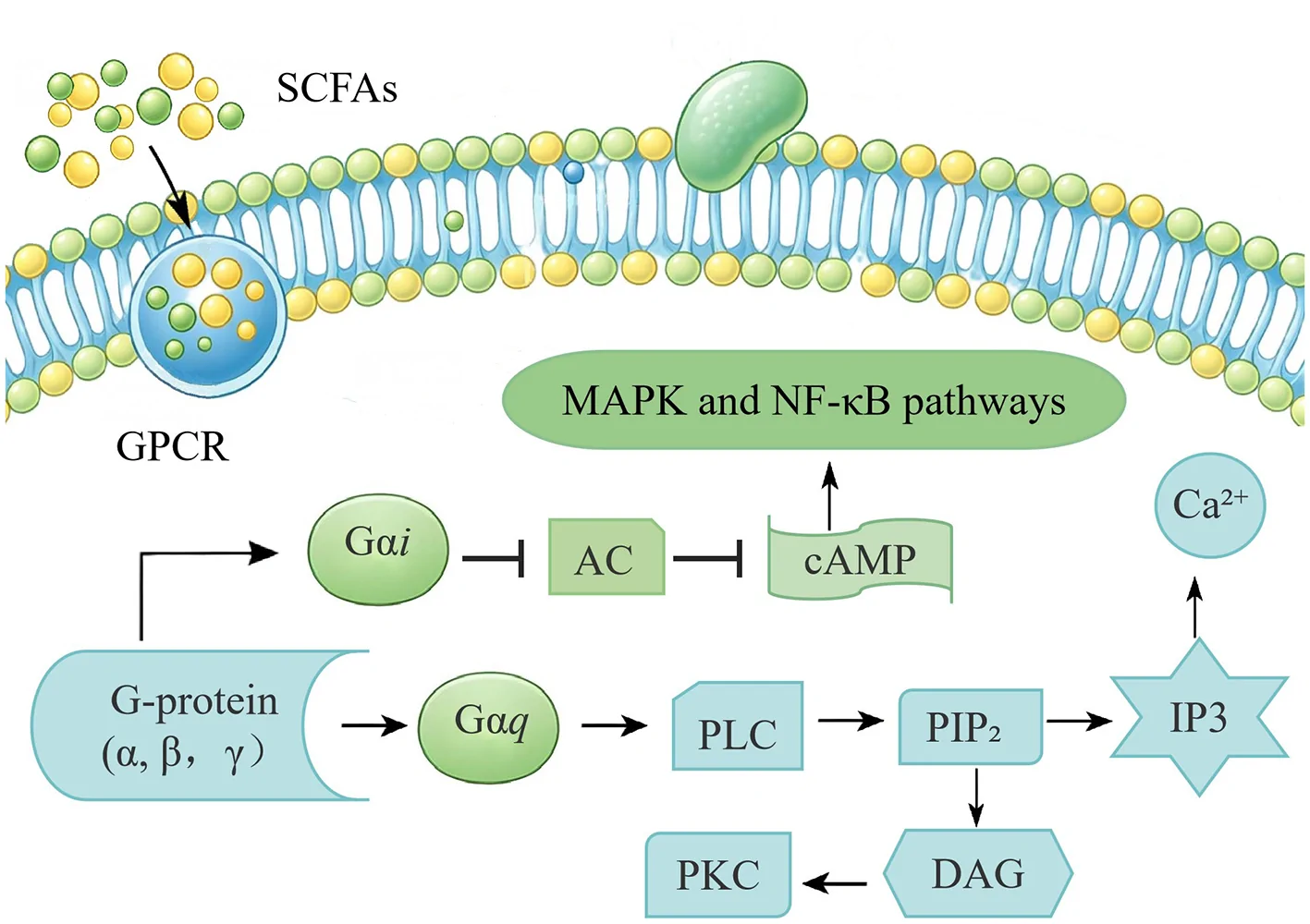
Immune-regulatory mechanisms of SCFAs through GPCR activation.

**Figure 3:**
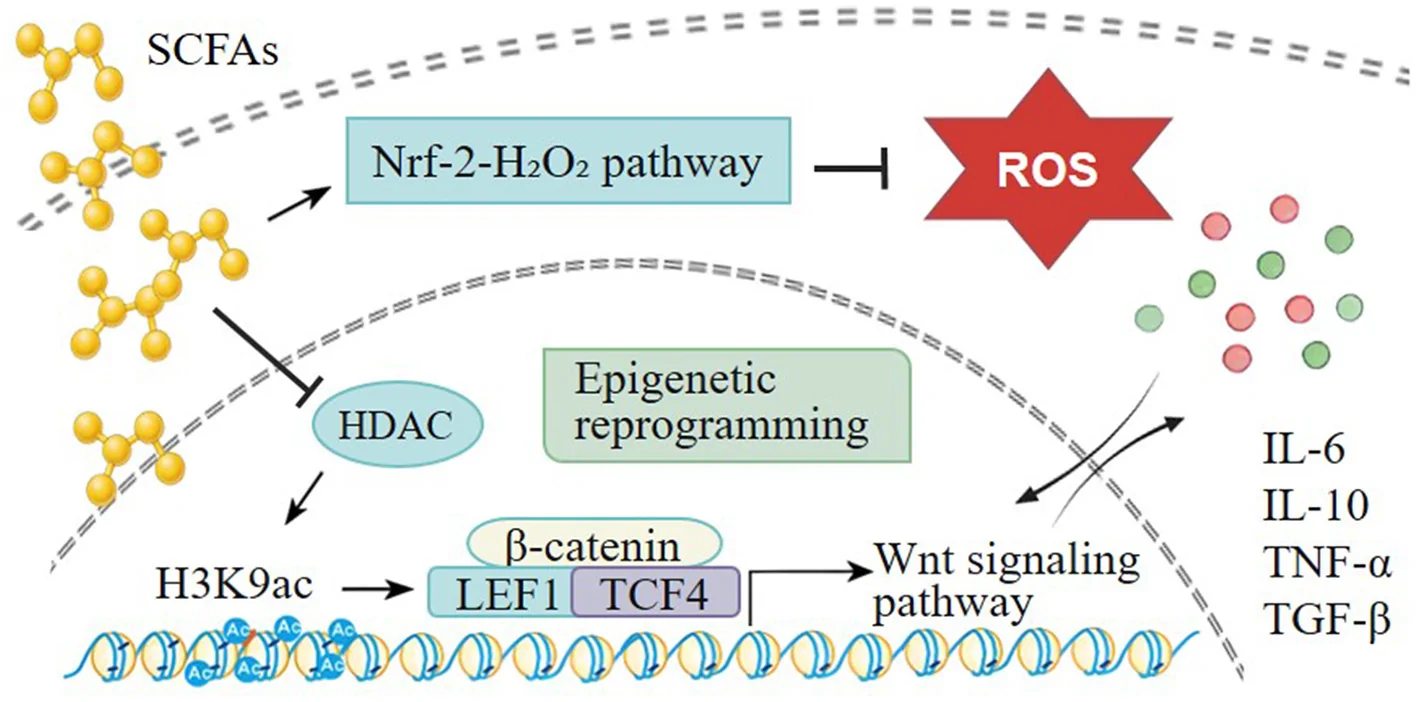
Immune-regulatory mechanisms of SCFAs through HDAC inhibition.

### 3.1 Regulation of myeloid cells

SCFAs are key regulators of myeloid cell function. At the macrophage level, Butyrate and propionate can significantly inhibit the polarization of M2 macrophages. They alleviate allergic airway inflammation in animal models of asthma and relieve asthma symptoms by increasing intestinal SCFA levels through fecal microbiota transplantation (46, 47). At the molecular level, SCFAs activate receptors such as GPR43, thereby inhibiting downstream cAMP production and regulating the MAPK/NF-κB pathways. This effectively suppresses the production of pro-inflammatory cytokines (including I L-1β, IL-6, and TNF-α) by myeloid cells and monocytes in response to stimuli such as lipopolysaccharide (LPS) (48). Furthermore, butyrate enhances the antibacterial effects of macrophages through HDAC3-mediated inhibition and can increase microbial clearance capacity by upregulating HIF-1α expression via HDAC inhibition (46, 49).

Regarding DCs, propionate inhibits the initiation of Th2 immune responses by DCs (46). SCFAs, on the other hand, generally suppress the production of IL-6 and TNF-α in myeloid DCs and plasmacytoid DCs (50). This mechanism not only relies on HDACs inhibition but also involves stimulating the SFK/PI3K/Rho family signaling pathway and activating actin polymerization, thereby promoting dendritic cell elongation and enhancing their antigen uptake and presentation capabilities (51).

### 3.2 Regulation of T lymphocytes

The regulation of T-cell differentiation and function by SCFAs is a central component of adaptive immunity. Their primary role lies in the regulation of the balance among CD4+ T-cell subsets. On the one hand, butyrate strongly promotes the differentiation of regulatory T cells (Tregs) and the production of IL-10, thereby maintaining immune homeostasis and suppressing inflammation (13, 52). On the other hand, SCFAs regulate Th17 cell development under specific conditions (13). At the same time, Butyrate and propionate can effectively suppress the production of effector cytokines such as TNF-α, IFN-γ, and IL-17 by activated T cells (50).

At the molecular level, these effects are achieved synergistically through three pathways. (1) GPCRs activation: SCFAs regulate signaling cascades within T cells via receptors such as GPR41 and GPR43 (53); (2) HDACs inhibition: SCFAs act as HDACs inhibitors, altering the expression of key genes through epigenetic modifications. For example, by inhibiting HDAC1, they upregulate Fas to induce apoptosis in activated T cells (13); (3) Metabolic reprogramming: butyrate can increase acetyl-CoA levels in T cells, activate mTOR signaling and fatty acid oxidation (FAO), thereby reprogramming T cell metabolism. This meets the unique energy and metabolic demands of effector and memory T cells (13, 37). Ultimately, these mechanisms collectively determine the protective or regulatory roles of SCFAs in antiviral and antitumor immunity, as well as in autoimmune diseases (54, 55).

### 3.3 Regulation of B lymphocytes and other immune cells

SCFAs also directly regulate the fate and function of B cells. By acting as HDACs inhibitors, butyrate and propionate alter the epigenetic state of B cells and reduce the expression of key genes such as Aicda and Prdm1, thereby influencing class-switch recombination (CSR) and plasma cell differentiation (56). Functionally, SCFAs stimulate B-cell activation and promote IgA production, while simultaneously reducing both total and antigen-specific IgE levels, demonstrating potential for alleviating allergic reactions (57). Interestingly, the effects of SCFAs on B cells are dose-dependent: lower doses (20 mm butyrate and 30 mm propionate) enhance CSR and somatic hyper-mutation, whereas higher dose (140 mm butyrate, and 150 mm propionate) inhibit them (56). Furthermore, SCFAs can influence the function of other innate immune cells, such as NK cells, through GPCR-dependent or non-GPCR-dependent pathways, collectively forming a regulatory network that shapes the overall immune landscape (3).

### 3.4 Biphasic regulation of concentration effects

Butyrate exhibits a typical biphasic effect on macrophages. At lower concentrations (0.1 mm), it inhibits production of pro-inflammatory cytokines such as TNF-α via the PPAR-γ signaling pathway and histone acetylation. In contrast, higher concentrations (10 mm) actually enhance the inflammatory response and induce cell death (58, 59). The molecular basis for this biphasic effect may lies in the fact that at lower concentrations, SCFAs primarily activate GPCR-mediated anti-inflammatory signaling, whereas at higher concentrations, HDAC-inhibitory effects predominate, leading to widespread epigenetic reprogramming (60, 61).

At the level of the intestinal epithelial barrier, SCFAs also exhibit concentration-dependent effects. Physiological concentrations (1–10 mm) promote tight junction assembly, whereas higher concentrations (≥50 mm) disrupt the barrier structure (62). In B cells, lower concentrations of SCFAs promote IgA and IgG production by upregulating AID and BLIMP-1 through HDACs inhibition, whereas higher concentrations suppress humoral immunity by upregulating miRNAs targeting Aicda and Prdm1 (63).

The key insight is that the concentration-dependent effects of SCFAs are not simply a matter of “the higher, the better” or “the lower, the better.” Future research should establish concentration-response curves for SCFA.

### 3.5 Cell type-specific regulation

Butyrate exerts distinctly different regulatory effects on CD4+ T cells and CD8+ T cells (64). In CD4+ T cells, butyrate enhances H3 acetylation at the Foxp3 locus through HDACs inhibition, thereby promoting Treg differentiation and suppressing Th17 cells (65). In CD8+ T cells, however, butyrate drives ID2-dependent upregulation of IL-12R by enhancing H3K9/14 acetylation at the Id2/Tbx21 promoters, thereby increasing the production of IFN-γ and granzyme B (63). This difference stems from the distinct baseline epigenetic landscapes of the different T cell subsets. The Foxp3 locus is in an “inducible” state in CD4+ T cells, but not in CD8+ T cells.

In macrophages, butyrate inhibits M2 polarization in the asthma microenvironment, whereas in the tumor microenvironment, it promotes M2 polarization and accelerates tumor progression (46). This seemingly contradictory phenomenon suggests that SCFAs do not directly “determine” the direction of macrophage polarization. Rather, they act as “modulators” based on existing polarization signals in the microenvironment. In a Th2-dominated asthma microenvironment, they amplify anti-inflammatory signals, whereas in a tumor-suppressive microenvironment, they are “hijacked” as pro-tumor signals.

The immunomodulatory effects of SCFAs are the product of the interaction between “intracellular programs” and “SCFA signals.” The baseline epigenetic states and signaling networks of different immune cells determine how they interpret the same SCFA signal. This suggests that future mechanistic studies should move beyond the linear paradigm of “SCFA → receptor → downstream signaling” and shift toward an interactive paradigm of “SCFA signal × intracellular state.”

## 4 The role of SCFAs in immune-related diseases

Gut microbiota dysbiosis is closely associated with the onset and progression of various immune-related diseases (66–68) (Table 2). As the primary metabolic products resulting from the fermentation of dietary fiber by the gut microbiota, SCFAs have emerged as a key molecular link between dysbiosis and host immunopathology (35, 69).

**Table 2:** Summary of research on SCFAs in immune-related diseases.

Disease/condition		Specific SCFA(s) and concentration	Models	Intervention duration	Key findings & proposed mechanisms	References
Cancer	Colorectal cancer	Butyrate 0.5–2 mm butyrate/propionate 0.5–2 mm	*In vitro*	24–72 h	Inducing cell cycle arrest and apoptosis; regulates chromatin landscape and ID2 expression via HDAC inhibition and p300/CBP-dependent epigenetic reprogramming; Inducing ROS production and reprograms metabolic profiles through metabolic reprogramming and ROS-mediated pathways	(71, 102)
	Breast cancer	Butyrate/propionate 0.5–5 mm	*In vitro*	24–72 h	Downregulating Cyclin A2, inducing cell cycle arrest and apoptosis via HDAC inhibition and cell-cycle regulation; Inhibiting migration and invasion by regulating EMT and suppressing MEK/ERK signaling	(31)
	HCC	SCFAs (general) endogenous/fecal microbiome	Animal/human	—	Exerting dual (pro- or anti-tumor) effects via gut–liver axis immune homeostasis and immune escape modulation; Associating with treatment response and survival, suggesting a prognostic biomarker role	(53, 76)
	Pancreatic cancer	Butyrate endogenous	Animal/human	—	Decreased levels correlate with carcinogenesis; Influencing cell signaling, autophagy, and cell cycle regulation	(74)
	Gastric cancer	Butyrate 0.5–5 mm	*In vitro*	24–72 h	Exerting anti-proliferative effects via HDAC inhibition and cell-cycle interference	(103)
	Multiple myeloma	SCFAs (general) 0.5–2 mm	*In vitro*	24 h	Enhancing NK cell cytotoxicity by inducing extracellular vesicle release and reducing IL-10 secretion	(75)
	CAR-T cell therapy	Butyrate 0.5–1 mm	*In vitro*	48 h	Enhancing antitumor activity, Promoting memory phenotype, and Reducing exhaustion markers via metabolic reprogramming (glycolysis + OXPHOS dual activation) and ECM remodeling	(74)
Autoimmune diseases	RA	Butyrate endogenous/ probiotic/high-fiber diet	Human/animal	12 weeks	Restoring Th17/Treg balance via AMPK/mTOR metabolic reprogramming, HDAC inhibition, and NLRP3 inflammasome suppression; Alleviating joint damage by inhibiting HDAC/NF-κB signaling; Reducing joint inflammation by modulating butyrate and rebalancing Treg/Tfr immunity; Lowering DAS28 by 1.8 points via raising SCFA levels and immunomodulation	(78, 104–106)
	T1D	Acetate 200 mm/humanized gnotobiotic	Animal	8 weeks	Ameliorating hyperglycemia and adipose loss by restoring gut microbiota structure and diversity; Delaying diabetes onset by enriching arginine, glutamate, and tryptophan metabolic pathways	(29, 79)
	MS	Propionate/Butyrate/SCFAs (general) endogenous	Animal/human	14 days	Reducing microglial activation and pro-inflammatory cytokines through HDAC1 inhibition and promotion of Ac-H3K9 expression; Reduced SCFA-producing bacteria correlate with disease progression; Influencing T-cell differentiation, mitochondrial function, and glial dynamics	(19)
	SLE	SCFAs (general) endogenous	Human/animal	—	Modulating autoantibody production via epigenetic regulation of B-cell function	(80, 81)
Metabolic diseases	T2DM	SCFAs (general) endogenous/mulberry leaf extract	Human/animal	8 weeks	Decreased butyrate correlates with insulin resistance; Acting through GPR41/GPR43 activation; Improving insulin resistance via SCFAs-AMPK/SIRT1/PGC-1α signaling and WAT browning	(82, 83)
	MASLD	Propionate	Animal	12 weeks	Attenuating hepatic inflammation and fibrosis through gut–liver axis (butyrate-TGR5-IκB-α/NF-κB signaling)	(86)
	CVD	SCFAs (general) endogenous	Human/animal	—	Associated with atherosclerosis, blood pressure, and myocardial inflammation via immune modulation, endothelial function, and lipid metabolism	(88, 107–110)
	IBD	SCFAs (general) endogenous	Animal/human	—	Protecting intestinal barrier and alleviates inflammation by inhibiting PI3K/AKT/mTOR and promoting autophagy	(87, 111)
**Neurological Diseases**	PD	Propionate/Butyrate	Animal	14 days	Modulating α-synuclein aggregation and neuroinflammation via GPR43-NLRP3 signaling; Attenuating neuroinflammation by inhibiting microglial HDAC1	(93)
	AD	Butyrate endogenous	Animal/human	—	Affecting amyloid formation and tau phosphorylation through epigenetic regulation and BBB maintenance	(94, 112)
	Autism spectrum disorder (ASD)	SCFAs (general) endogenous	Animal/human	—	Influencing neurodevelopment via gut–brain axis through immune, endocrine, and neural pathways	(95, 96)
**Allergic Diseases**	Allergic airway inflammation	SCFAs (general) dietary fiber	Animal	—	Reducing allergic inflammation by modulating ILC2 activity	(99)
	Atopic dermatitis	Butyrate endogenous	Animal	—	Improving AD pathology via GPR109A activation and inhibition of IκB-α, p-NF-κB, and p-JNK	(100, 101)

Dose ranges are typical *in vitro* concentrations. *In vivo* doses vary by route and model – refer to original publications for details. Evidence levels differ by study design (human/animal/*in vitro*). This table aims to present the full landscape of evidence, not to grade it.

### 4.1 Tumor immunology

SCFAs exert bidirectional effects in tumors. In colorectal cancer, butyrate exhibits a “paradoxical effect”: it promotes proliferation in normal intestinal epithelium, while in cancer cells, it induces cell cycle arrest and apoptosis through HDAC inhibition (70). Some studies have also found that the effects of butyrate on gene expression are independent of HDAC inhibition and instead depend on p300/CBP. This suggests that gene programs may be regulated through histone butyration (71). Regarding immune checkpoint regulation, SCFAs can also promote the activation and infiltration of CD8+ T cells and the production of interferon-γ (IFN-γ) through the transcription regulator ID2, thereby enhancing antitumor effects (13, 72). It also activate GPR81 signaling, which leads to PD-L1 overexpression and reduces the antigen-presenting capacity of dendritic cells (73). In CAR-T therapy, butyrate reshapes the metabolic adaptation of CAR-T cells through dual activation of glycolysis and oxidative phosphorylation, inducing a memory-like phenotype and reducing markers of exhaustion. At the same time, it upregulates extracellular matrix (ECM)-remodeling genes (MMPs, Collagens), thereby improving tumor homing capacity (74). In other tumors, butyrate and propionate exert anti-proliferative and anti-invasive effects by downregulating Cyclin A2, modulating EMT, and inhibiting MEK/ERK (31). SCFAs also enhance NK cell cytotoxicity (75). In hepatocellular carcinoma, SCFAs exhibit bidirectional effects and are associated with response to TACE therapy (53, 76).

### 4.2 Autoimmune diseases

The protective role of SCFAs in autoimmune diseases is no longer limited to “restoring the Th17/Treg balance.” In rheumatoid arthritis (RA), SCFAs exert their effects through a triple synergistic mechanism: AMPK/mTOR metabolic reprogramming, HDAC inhibition, and NLRP3 inflammasome inhibition (13, 77). Butyrate alleviates joint damage by inhibiting HDAC/NF-κB signaling and may indirectly regulate the invasive phenotype of fibroblast-like Synovial cells (FLS) by influencing their proliferation, migration, and MMP secretion (127). The butyrate-producing bacterium *Eubacterium rectale* alleviates joint inflammation by regulating butyrate levels and restoring the Treg/Tfr balance (78). In type 1 diabetes, acetate restores gut microbiota structure and diversity. SCFA-based biologic therapy delays the onset of diabetes by enriching arginine, glutamate, and tryptophan metabolic pathways (29, 79). In multiple sclerosis (MS), propionate and butyrate alleviate neuro-inflammation by inhibiting HDAC1 and promoting Ac-H3K9 expression (19). A reduction in SCFA-producing bacteria is associated with disease progression (106). In systemic lupus erythematosus, SCFAs regulate autoantibody production through epigenetic regulation of B-cell function (80, 81).

### 4.3 Metabolic diseases and inflammatory bowel disease

In type 2 diabetes, SCFAs alleviate inflammation and insulin resistance by activating GPR41/GPR43 and promote browning of white adipose tissue (WAT) through AMPK/SIRT1/PGC-1α signaling (82, 83). In MASLD, *Clostridium butyricum* supplementation alleviates liver inflammation and fibrosis via the butyrate-TGR5-IκB-α/NF-κB signaling pathway through the gut-liver axis (84–86). In inflammatory bowel disease, SCFAs promote colonic autophagy and protect the intestinal barrier by inhibiting the PI3K/AKT/mTOR pathway (87). In cardiovascular disease, SCFAs are involved in the regulation of atherosclerosis, blood pressure, and myocardial inflammation (88).

### 4.4 Neurological disorders

SCFAs regulate neuro-inflammation, blood-brain barrier integrity, and neurotransmitter systems via the “gut-brain axis” (6, 89–92). In Parkinson's disease (PD), SCFAs regulate α-synuclein aggregation and neuro-inflammation via the GPR43-NLRP3 signaling pathway and influence microglial activation and oxidative stress (93). In Alzheimer's disease (AD), butyrate influences epigenetic regulation, neuro-inflammation, blood-brain barrier maintenance, amyloid formation, and tau phosphorylation (94). In autism spectrum disorder (ASD), the gut-brain axis influences neurodevelopment and behavioral symptoms through immune, endocrine, and neural pathways (95, 96).

### 4.5 Allergic diseases

SCFAs regulate allergic immune responses by acting on Th2 cells, ILC2s, eosinophils, mast cells, and basophils (97). The levels of SCFA in early life are associated with a protective effect against atopic dermatitis, asthma, and food allergies (98, 99). Butyrate improves atopic dermatitis by activating GPR109A (100). *Bifidobacsterium* FB3-12 alleviates ovalbumin-induced allergies by enhancing intestinal barrier function and upregulating SCFA and related GPR levels (101).

The association between SCFAs and disease suggests that they may serve as potential targets for intervention. However, observational studies typically only suggest associations and cannot establish causality. Decreased SCFA levels may result from disease states, dietary changes, or gut microbiota dysbiosis. Furthermore, results from animal and *in vitro* studies cannot be directly extrapolated to clinical settings. Future research should further validate causal relationships through longitudinal cohort studies, Mendelian randomization, randomized controlled trials, and mechanistic intervention studies.

### 4.6 Microenvironment dependency

The effects of SCFAs in the tumor microenvironment (TME) are markedly different from those in steady-state tissues. In the TME, SCFAs can, on the one hand, enhance the metabolic adaptability and cytotoxicity of CD8+ T cells and promote M1 macrophage polarization to enhance antitumor immunity. On the other hand, they may weaken the efficacy of immunotherapy by activating Tregs or inhibiting antigen presentation by DCs. At the core of this bidirectional immune regulation lie metabolic competition, hypoxia, and the acidic environment present in the TME. These factors alter the metabolic state and signaling pathway activity of immune cells, thereby modifying the effects of SCFAs (113).

In IBD, the role of SCFAs is similarly controversial. SCFAs exert a protective effect by promoting Treg proliferation, suppressing the Th17 axis, and improving the intestinal barrier (113–115). However, higher concentrations of butyrate (>5 mm) can activate the NLRP3 inflammasome in macrophages and increase IL-1β release (116). Under conditions of dysbiosis, although acetate and propionate exert anti-inflammatory effects via GPR43 under steady-state conditions, they can exacerbate Th1/Th17 responses in models of dysbiotic colitis (116).

It is evident, therefore, that SCFAs are not fixed “anti-inflammatory molecules” or “pro-inflammatory molecules”. Rather, the microenvironment reshapes the role of short-chain fatty acids by altering the “signal interpretation system” of immune cells.

### 4.7 Paradoxical effects in the context of disease

SCFAs may exert diametrically opposed effects in different diseases or even at different stages of the same disease. Taking cancer as an example, butyrate inhibits cell motility and Akt/ERK signaling in colorectal cancer through an HDAC3-dependent mechanism (114). Butyrate can enhance the suppression of lung cancer through the HDAC1/ID2/IL-12R pathway, lysosome Fe^2+^- and SLC7A11-mediated ferroptosis (117, 118). However, butyrate may promote the proliferation of lung cancer cells at low concentrations (1 mm), while a high concentration (5 mm) of butyrate suppressed it. Treatment of a C57BL/6 mouse LLC tail-vein metastasis model with a lower-concentration (40 mm) butyrate aerosol resulted in a significant increase in both the number of lung metastases and the maximum volume of lung metastases. The number of lung metastases in the higher-concentration butyrate group (200 mm) was similar to that in the saline group (119). In immune-therapy, high fecal SCFA levels are associated with prolonged progression-free survival following anti-PD-1 treatment. However, in anti-CTLA-4 therapy, they may attenuate treatment efficacy by inhibiting ICOS signaling (120). These treatment-specific differences suggest that the effects of SCFAs depend not only on concentration but also on the type of immune checkpoint pathway activated.

In autoimmune diseases, SCFAs generally exert a protective effect by promoting Tregs and suppressing Th17 cells (61). However, SCFAs can also enhance the function of effector T cells and B cells (62, 63, 114, 115). This suggests that under specific conditions, SCFAs may exacerbate autoimmunity. This “protective vs. pathogenic” switch may depend on the stage of the disease and whether local SCFA concentrations exceed a certain threshold. This “context-dependence” suggests that future therapeutic strategies should not merely aim to “increase short-chain fatty acid levels,” but rather focus on “targeting the right cells in the right tissues, at the right time, and at the right concentration.”

## 5 Targeted regulation strategies for SCFAs

SCFAs play a central role in immune regulation and inflammation control, and imbalances in their levels are closely associated with various diseases. Therefore, targeted intervention strategies for SCFAs have become an important area of focus in both basic research and clinical translation. Currently, mainstream regulatory approaches primarily encompass three categories: dietary intervention, probiotic/prebiotic supplementation, and fecal microbiota transplantation. These strategies aim to restore or enhance endogenous SCFA production, thereby reestablishing immune homeostasis.

### 5.1 Dietary fiber and nutritional intervention

Dietary fiber serves as a substrate for the production of SCFAs; its intake directly influences the production and composition of SCFAs in the gut, which in turn affects host health (121, 122). Different dietary components have distinct effects. Saturated fatty acids promote inflammation and reduce the abundance of SCFA-producing bacteria, while n-3 polyunsaturated fatty acids have anti-inflammatory effects and promote their enrichment (123). Therefore, optimizing dietary composition can both increase SCFA levels and modulate the composition and metabolic functions of the gut microbiota.

Notably, SCFAs exert systemic effects via the “gut-X axis”. SCFAs derived from dietary fiber are key drivers of the gut-bone axis and influence bone remodeling (16, 85). In the gut-lung axis, they reduce ischemia-reperfusion-induced IL-1β release and regulate pulmonary immune tone, suggesting their potential for intervention in pulmonary inflammation (124). Dietary fiber from coconut co-products increases intestinal SCFAs, preventing overweight, dyslipidemia, and oxidative damage in rats (125). Fiber deficiency disrupts colonic homeostasis and induces anxiety-like behavior via the gut-brain axis. Inulin supplementation restores the intestinal barrier, increases SCFAs levels, upregulates FFAR2/FFAR3, and, via the TLR4/MyD88/NF-κB pathway, reduces LPS levels and inhibits hippocampal neuro-inflammation (126).

Dietary fiber intervention is a fundamental strategy for increasing SCFAs levels. It is safe, cost-effective, and scalable. However, excessive intake can lead to bloating and impaired mineral absorption. Future research should focus on standardizing fiber classification, elucidating the mechanisms underlying individual differences, and studying multi-organ pharmacokinetics.

This will advance dietary interventions from “empirical recommendations” to “precision prescriptions”.

### 5.2 Probiotic, prebiotic, and postbiotic interventions

Probiotics are live microorganisms. When consumed in sufficient quantities, they can promote health by modulating the immune system and repairing the intestinal barrier (97). Traditional probiotics, such as *Lactobacillus* and *Bifidobacterium*, promote the production of SCFAs and play a role in various diseases (127). Supplementation during childhood is more effective at increasing fecal SCFAs and IgA levels (128). Probiotic intervention can also prolong the survival of hamsters infected with scabies mites and alleviate neuropathological damage (129). *Akkermansia muciniphila*, a next-generation probiotic, increases SCFAs in the liver. When combined with butyrate, it has shown therapeutic potential in diabetic models (130). A specific combination (*Lacticaseibacillus rhamnosus* HN001, *Bifidobacterium lactis* HN019, and *Lactobacillus acidophilus* NCFM) alleviates allergies by suppressing Th2 responses, reducing IgE, histamine, and pro-inflammatory factors, and enhancing Treg activity and TGF-β1 expression (131).

Prebiotics, as selectively fermentable dietary components, can reduce inflammation and influence immune responses by modulating the gut microbiota-estrogen axis. In breast cancer, prebiotics demonstrate anticancer potential by targeting estrogen receptors and the PI3K/AKT/mTOR pathway via SCFAs, thereby regulating estrogen metabolism, immune function, and metabolic pathways (132). Prebiotic chronotherapy alleviates depression-like behaviors in FMT mice by enhancing SCFA receptors and the intestinal barrier (133). Microbiome interventions, including probiotics, prebiotics, synbiotics, and postbiotics, can modulate the host's innate and adaptive immune responses (134). As a core strategy targeting SCFAs, these interventions are mild in effect, highly safe, and suitable for long-term use.

However, this field still faces challenges such as strain specificity vs. universal effects, colonization efficiency vs. sustainability of intervention. Achieving sustainable SCFAs regulation through synbiotic combinations, continuous administration, or screening for highly colonizing strains remains a bottleneck for translational applications.

### 5.3 Fecal microbiota transplantation

FMT is emerging as a new approach for gut microbiota intervention (135, 136). In T1D humanized mice, donor microbiota with SCFAs-related characteristics can delay the progression of diabetes, accompanied by human-like changes in metabolic pathways, mucosal immunity, and IgA (79). This suggests that SCFA-related immune regulation may be involved. In colitis, FMT enhances microbial diversity, density, and persistence, restoring Treg/gut homeostasis through TGF-β, IL-10^+^ APCs, and barrier reinforcement (52). These effects involve extensive immune remodeling, with SCFAs likely serving as one of the mediators. In neurological diseases, FMT may restore the microbiota, alleviate symptoms, and provide neuroprotection (137). In portal hypertension associated with cirrhosis, the benefits of FMT are associated with butyrate-mediated HDAC3 inhibition and activation of the PI3K/Akt/eNOS pathway (36). The traditional Chinese medicine “Zhenqi Fuzheng Granules” enhances the efficacy of PD-1 antibodies via the gut microbiota–SCFAs–GPR109A axis, supporting the potential of SCFAs-related mechanisms (138).

However, FMT is a multicomponent, multi-target intervention and is not a SCFA-specific strategy. In addition to altering SCFAs-producing bacteria and the level of SCFAs, it also affects bile acids, aryl receptor ligands, neurotransmitters, endotoxins, phages, and various immune pathways. Therefore, it remains unclear whether the beneficial effects of FMT stem from SCFAs or other factors. SCFAs may play a significant role, or they may merely be a concomitant phenomenon. There is currently no causal evidence.

### 5.4 Combined strategies and multi-target interventions

Given the complexity of the SCFA regulatory network, comprehensive interventions offer synergistic benefits. Probiotics, prebiotics, and FMT modulate the gut microbiota, thereby influencing pulmonary homeostasis and immunity. This provides a new approach for improving the prognosis of pulmonary diseases, including SARS-CoV-2 infection (139). *Lactobacillus gasseri*, galacto-oligosaccharides, FMT, and traditional Chinese medicine (TCM) (astragalus, curcumin) can enhance antiviral cytokines, restore gut-lung balance, and alleviate pulmonary inflammation (140). In terms of mental health, probiotics can alleviate depression and moderately reduce anxiety. Prebiotics show a positive trend in alleviating depression (141). Their use in combination with medication holds clinical promise. In autism spectrum disorder (ASD), increasing the abundance of Bifidobacterium can improve gastrointestinal symptoms (142). Probiotics, prebiotics, synbiotics, and FMT can alleviate ASD symptoms (95, 142–144). The “gut-first” paradigm for Parkinson's disease supports gut-targeted therapies such as probiotics, prebiotics, and FMT, for which preclinical and clinical evidence is available (145). Probiotics and bioactive compounds (e.g., berberine, polysaccharides) can strengthen the intestinal barrier, regulate metabolites such as SCFAs, and reduce inflammation (68).

As an emerging approach to modulating host immunity and inflammation, intervention strategies targeting SCFAs hold promise as safe and effective treatments for immune-related diseases (136). Although SCFAs interventions show great promise, they still face challenges. Individual differences in the microbiome lead to highly heterogeneous responses, highlighting the need for precision medicine (146, 147). Most studies are limited to the preclinical stage, and there is a lack of large-scale, rigorous clinical trials (137). Standardization of intervention protocols (strain, dose, route, and duration) still requires optimization (78, 148).

## 6 Perspective

SCFAs have been redefined central signaling molecules linking the gut microbiota and the host immune system from mere sources of intestinal energy. This review outlines the complex network through which SCFAs maintain immune homeostasis via multi-target, multi-pathway, and multi-level mechanisms. Mechanistically, SCFAs broadly regulate the functions of innate and adaptive immune cells through three pathways: GPCRs activation, HDACs inhibition and metabolic reprogramming. At the systemic level, SCFAs facilitate communication between local immunity and systemic homeostasis through multiple organ axes, including gut-liver, gut-brain, gut-lung, gut-joint, and gut-heart axes. In terms of disease and intervention, SCFA dysregulation is closely associated with autoimmune, metabolic, neoplastic, neurodegenerative, and allergic diseases. Dietary fiber, probiotics/prebiotics, fecal microbiota transplantation, and combined strategies constitute a multi-tiered intervention system ranging from basic to precision medicine.

Despite significant progress, this field still faces several key challenges: (1) A systematic lack of concentration-effect curves: The vast majority of *in vitro* studies use supraphysiological concentrations (in the mm range), whereas SCFA concentrations in the peripheral circulation are only in the μm range (30). Currently, there is a lack of complete dose-effect curves spanning physiological to pathological concentrations, making it impossible to determine the precise thresholds for functional shifts. (2) Unclear molecular basis of cell-type-specific mechanisms: Why does the same SCFA produce opposite effects in CD4+ and CD8+ T cells? The underlying differences in epigenetic and transcriptional regulation have not yet been systematically elucidated. (3) Unclear mechanisms by which micro-environmental signals “reprogram” SCFA effects: How does the tumor microenvironment transform SCFAs from “antitumor” to “tumor-promoting”? This involves multilevel interactions among metabolism, signaling pathways, and epigenetics, and there is currently a lack of integrated research. (4) The intermediary mechanisms of the gut-organ axis remain unclear: Do SCFAs act directly on distant organs via the circulation, or do they first modulate gut immune cells and then transmit their effects indirectly through migration or cytokines? Distinguishing between these two pathways is crucial for understanding the systemic immune-regulatory effects of SCFAs. (5) Gaps in the Synergistic Effects of Multiple SCFAs: Existing studies have largely focused on individual SCFAs, whereas SCFAs exist in a mixed form *in vivo*. Do different SCFAs act synergistically, antagonistically, or independently? Systematic research on this topic is currently lacking.

Looking ahead, at the mechanistic level, we should leverage single-cell sequencing and spatial metabolomics to analyze the spatiotemporal specificity of SCFAs, elucidate the molecular basis of the “paradoxical effect,” and map the quantitative transport profile of the “gut-X axis”. At the technical level, we need to develop multi-omics biomarkers to predict individual responses. At the translational level, we should advance large-scale randomized controlled trials to explore synergistic strategies between SCFAs and conventional drugs, as well as “therapeutic windows” for different diseases. At the application level, we should develop targeted delivery systems (such as nano-encapsulation) and deepen modern research on how active ingredients in traditional Chinese medicine regulate SCFAs.

In summary, the immune-regulatory network of SCFAs is transitioning from basic mechanisms to clinical translation. A deeper understanding of this network not only helps reveal the common pathological basis of various chronic diseases but also opens up new avenues for precision interventions based on microbial metabolites. This holds promise for breakthroughs in the prevention and treatment of autoimmune, metabolic, oncological, and neurodegenerative diseases.
